# Asymptotic tests for Hardy–Weinberg equilibrium in hexaploids

**DOI:** 10.1093/hr/uhac104

**Published:** 2022-05-17

**Authors:** Jing Wang, Li Feng, Shuaicheng Mu, Ang Dong, Jinwen Gan, Zhenying Wen, Juan Meng, Mingyu Li, Rongling Wu, Lidan Sun

**Affiliations:** Center for Computational Biology, College of Biological Sciences and Technology, Beijing Forestry University, Beijing 100083, China; Center for Computational Biology, College of Biological Sciences and Technology, Beijing Forestry University, Beijing 100083, China; Center for Computational Biology, College of Biological Sciences and Technology, Beijing Forestry University, Beijing 100083, China; Center for Computational Biology, College of Biological Sciences and Technology, Beijing Forestry University, Beijing 100083, China; Center for Computational Biology, College of Biological Sciences and Technology, Beijing Forestry University, Beijing 100083, China; Beijing Key Laboratory of Ornamental Plants Germplasm Innovation & Molecular Breeding, National Engineering Research Center for Floriculture, Beijing Laboratory of Urban and Rural Ecological Environment, School of Landscape Architecture, Beijing Forestry University, Beijing 100083, China; Beijing Key Laboratory of Ornamental Plants Germplasm Innovation & Molecular Breeding, National Engineering Research Center for Floriculture, Beijing Laboratory of Urban and Rural Ecological Environment, School of Landscape Architecture, Beijing Forestry University, Beijing 100083, China; Beijing Key Laboratory of Ornamental Plants Germplasm Innovation & Molecular Breeding, National Engineering Research Center for Floriculture, Beijing Laboratory of Urban and Rural Ecological Environment, School of Landscape Architecture, Beijing Forestry University, Beijing 100083, China; Center for Computational Biology, College of Biological Sciences and Technology, Beijing Forestry University, Beijing 100083, China; Center for Statistical Genetics, Departments of Public Health Sciences and Statistics, The Pennsylvania State University, Hershey, PA 17033, USA; Beijing Key Laboratory of Ornamental Plants Germplasm Innovation & Molecular Breeding, National Engineering Research Center for Floriculture, Beijing Laboratory of Urban and Rural Ecological Environment, School of Landscape Architecture, Beijing Forestry University, Beijing 100083, China

## Abstract

Hexaploids, a group of organisms containing three complete sets of chromosomes in a single nucleus, are of utmost importance to evolutionary studies and breeding programs. Many studies have focused on hexaploid linkage analysis and QTL mapping in controlled crosses, but little methodology has been developed to reveal how hexaploids diversify and evolve in natural populations. We formulate a general framework for studying the pattern of genetic variation in autohexaploid populations through testing deviation from Hardy–Weinberg equilibrium (HWE) at individual molecular markers. We confirm that hexaploids cannot reach exact HWE but can approach asymptotic HWE at 8–9 generations of random mating. We derive a statistical algorithm for testing HWE and the occurrence of double reduction for autopolyploids, a phenomenon that affects population variation during long evolutionary processes. We perform computer simulation to validate the statistical behavior of our test procedure and demonstrate its usefulness by analyzing a real data set for autohexaploid chrysanthemum. When extended to allohexaploids, our test procedure will provide a generic tool for illustrating the genome structure of hexaploids in the quest to infer their evolutionary status and design association studies of complex traits.

## Introduction

Polyploidy has been thought to be a driving force for evolution and speciation, especially in higher plants [1–4], but the genetic mechanisms underlying its evolution have not been fully understood [5]. One approach for studying genetic variation is to test for Hardy–Weinberg equilibrium (HWE) in a natural population, which predicts that gene frequencies and genotype frequencies stay constant after an episode of random mating if no evolutionary forces act in the population [6–8]. Since the discovery of HWE by early geneticists, its test has been indispensable for inferring diploid population variation [9–12]. The HWE test has also served as the basis for genetic association studies [5] and as a tool to monitor genotyping errors [13,14]. However, there is little literature on the use of the HWE test to study genetic variation in polyploid populations [15,16].

More recently, Sun et al. [17] developed a mathematical equation for characterizing how tetraploids approach HWE and implemented a statistical algorithm for testing the significance of deviation from HWE in a tetraploid population. Although the tetraploid model of Sun et al. can be straightforwardly extended to the study of hexaploids, this extension is exponentially more complex with increasing ploidy level, well deserving of articulation in a separate article. Dating back to the late 1940s, through a series of complicated mathematical derivations, Geiringer [18] found that, like tetraploids, hexaploids gradually reach population equilibrium. Yet, the author did not specifically visualize the asymptotic process of hexaploid HWE and, more importantly, because of the low computational proficiency in his time, he was not able to provide an algorithm for a hexaploid HWE test.

In this article, we formulate a procedure for monitoring and testing hexaploid HWE using widely available SNP markers. Unlike diploids in which three genotypes can be distinguished from one another for biallelic SNPs, polyploid heterozygotes characterized by most existing sequencing techniques are genotype-ambiguous or dosage-unknown, providing a limited amount of information for population genetic analysis. The complexity of identifying meiotic behavior based on dosage-unknown genotypes dramatically increases with ploidy level. The double reduction of autopolyploids, a meiotic phenomenon by which two sister chromatids of a single chromosome segregate into the same gamete after crossover [19], which is believed to shape the evolutionary consequences of organisms [1], increases this complexity. Our testing procedure is generic, accommodating genotype ambiguity and double reduction. We perform computer simulation to examine the statistical properties of our procedure and validate its utility by analyzing data from autohexaploid chrysanthemum.

## Model

**How do autohexaploid genotypes segregate and transmit from parental to offspring generations?** Consider a SNP marker with two alleles *A* and *a*, which form four possible triploid gametes, *AAA*, *AAa*, *Aaa*, and *aaa*, in a hexaploid population. Through random unification, these gametes form seven possible genotypes, *AAAAAA* (6A), *AAAAAa* (5A1a), *AAAAaa* (4A2a), *AAAaaa* (3A3a), *AAaaaa* (2A4a), *Aaaaaa* (1A5a), and *aaaaaa* (6a). These genotypes will produce triploid gametes with different frequencies determined by both Mendel’s first law and double reduction for autohexaploids. Table 1 lists the gamete frequencies from each parental genotype. For purely homozygous genotypes *AAAAAA* or *aaaaaa*, the same gamete type is identified, although its formation results from either double reduction or non-double reduction.

**Table 1:** Gamete frequencies derived from a parental genotype in an autohexaploid population

Gamete Frequency				
Genotype	*AAA*	*AAa*	*Aaa*	*aaa*
6*A*	1	0	0	0
5*A*1*a*	1/2 + 1/6α	1/2–1/3α	1/6α	0
4*A*2*a*	1/5 + 1/5α	3/5–1/3α	1/5 + 1/15α	1/15α
3*A*3*a*	1/20 + 3/20α	9/20–3/20α	9/20–3/20α	1/20 + 3/20α
2*A*4*a*	1/15α	1/5 + 1/15α	3/5–1/3α	1/5 + 1/5α
1*A*5*a*	0	1/6α	1/2–1/3α	1/2 + 1/6α
6*a*	0	0	0	1

We use *P*_6A_(*t*−1), *P*_5A1a_(*t*−1), *P*_4A2a_(*t*−1), *P*_3A3a_(*t*−1), *P*_2A4a_(*t*−1), *P*_1A5a_(*t*−1), and *P*_6a_(*t*−1) to denote the frequencies of the seven hexaploid genotypes in the parental population (generation *t*−1). Gametes derived from each parental genotype combine randomly between parents to generate the offspring genotypes, with frequencies that depend on the frequencies of mating types and Table 1’s gamete frequencies (Table 2). Let *P*_6A_(*t*), *P*_5A1a_(*t*), *P*_4A2a_(*t*), *P*_3A3a_(*t*), *P*_2A4a_(*t*), *P*_1A5a_(*t*), and *P*_6a_(*t*) denote the corresponding genotype frequencies in the offspring population (generation *t*), with forms given in Supplemental Text 1.

**Table 2:** The frequencies of offspring genotypes derived from each mating type of parents in a natural panmictic autohexaploid population

Parental Mating		Offspring Generation						
Type	Frequency	6*A*	5*A*1*a*	4*A*2*a*	3*A*3*a*	2*A*4*a*	1*A*5*a*	6*a*
6*A* × 6*A*	*P*_6*A*_^2^(*t*−1)	1	0	0	0	0	0	0
6*A* × 5*A*1*a*	2*P*_6*A*_(*t*−1)*P*_5*A*1*a*_(*t*−1)	1/2 + 1/6*α*	1/2–1/3*α*	1/6*α*	0	0	0	0
6*A* × 4*A*2*a*	2*P*_6*A*_(*t*−1)*P*_4*A*2*a*_(*t*−1)	1/5 + 1/5*α*	3/5–1/3*α*	1/5 + 1/15*α*	1/15*α*	0	0	0
6*A* × 3*A*3*a*	2*P*_6*A*_(*t*−1)*P*_3*A*3*a*_(*t*−1)	1/20 + 3/20*α*	9/20–3/20*α*	9/20–3/20*α*	1/20 + 3/20*α*	0	0	0
6*A* × 2*A*4*a*	2*P*_6*A*_(*t*−1)*P*_2*A*4*a*_(*t*−1)	1/15*α*	1/5 + 1/15*α*	3/5–1/3*α*	1/5 + 1/5*α*	0	0	0
6*A* × *1A5a*	2*P*_6*A*_(*t*−1)*P*_1*A*5*a*_(*t*−1)	0	1/6*α*	1/2–1/3*α*	1/2 + 1/6*α*	0	0	0
6*A* × 6*a*	2*P*_6*A*_(*t*−1)*P*_6*a*_(*t*−1)	0	0	0	1	0	0	0
5*A*1*a* × 5*A*1*a*	*P*_5*A*1*a*_^2^(*t*−1)	1/4 + 1/36α^2^ + 1/6α	1/2–1/9α^2^–1/6α	1/4 + 1/6α^2^–1/6α	–1/9α^2^ + 1/6α	1/36α^2^	0	0
5*A*1*a* × 4*A*2*a*	2*P*_5*A*1*a*_(*t*−1)*P*_4*A*2*a*_(*t*−1)	1/10 + 1/30α^2^ + 2/15α	2/5–11/90α^2^–1/30α	2/5 + 7/45α^2^–4/15α	1/10–1/15α^2^ + 1/10α	–1/90α^2^ + 1/15α	1/90α^2^	0
5*A*1*a* × 3*A*3*a*	2*P*_5*A*1*a*_(*t*−1)*P*_3*A*3*a*_(*t*−1)	1/40 + 1/40α^2^ + 1/12α	1/4–3/40α^2^ + 7/120α	9/20 + 1/20α^2^–13/60α	1/4 + 1/20α^2^–1/15α	1/40–3/40α^2^ + 2/15α	1/40α^2^ + 1/120α	0
5*A*1*a* × 2*A*4*a*	2*P*_5*A*1*a*_(*t*−1)*P*_2*A*4*a*_(*t*−1)	1/90α^2^ + 1/30α	1/10–1/90α^2^ + 1/10α	2/5–1/15α^2^–1/10α	2/5 + 7/45α^2^–1/5α	1/10–11/90α^2^ + 2/15α	1/30α^2^ + 1/30α	0
5*A*1*a* × 1*A*5*a*	2*P*_5*A*1*a*_(*t*−1)*P*_1*A*5*a*_(*t*−1)	0	1/36α^2^ + 1/12α	1/4–1/9α^2^	1/2 + 1/6α^2^–1/6α	1/4–1/9α^2^	1/36α^2^ + 1/12α	0
5*A*1*a* × 6*a*	2*P*_5*A*1*a*_(*t*−1)*P*_6*a*_(*t*−1)	0	0	0	1/2 + 1/6*α*	1/2–1/3*α*	1/6*α*	0
4*A*2*a* × 4*A*2*a*	*P*_4*A*2*a*_^2^(*t*−1)	1/25 + 1/25α^2^ + 2/25α	6/25–2/15α^2^ + 8/75α	11/25 + 31/225α^2^–22/75α	6/25–4/225α^2^–2/75α	1/25–9/225α^2^ + 8/75α	2/225α^2^ + 2/75α	1/225α^2^
4*A*2*a* × 3*A*3*a*	2*P*_4*A*2*a*_(*t*−1)*P*_3*A*3*a*_(*t*−1)	1/100 + 3/100α^2^ + 1/25α	3/25–2/25α^2^ + 2/15α	37/100 + 3/100α^2^–11/75α	37/100 + 2/25α^2^–59/300α	3/25–7/100α^2^ + 31/300α	1/100 + 19/300*α*	1/100α^2^ + 1/300α
4*A*2*a* × 2*A*4*a*	2*P*_4*A*2*a*_(*t*−1)*P*_2*A*4*a*_(*t*−1)	1/75α^2^ + 1/75α	1/25–2/225α^2^ + 7/75α	6/25–19/225α^2^ + 1/25α	11/25 + 4/25α^2^–22/75α	6/25–19/225α^2^ + 1/25α	1/25–2/225α^2^ + 7/75α	1/75α^2^ + 1/75α
4*A*2*a* × 1*A*5*a*	2*P*_4*A*2*a*_(*t*−1)*P*_1*A*5*a*_(*t*−1)	0	1/30α^2^ + 1/30α	1/10–11/90α^2^ + 2/15α	2/5 + 7/45α^2^–1/5α	2/5–1/15α^2^–1/10α	1/10–1/90α^2^ + 1/10α	1/90α^2^ + 1/30α
4*A*2*a* × 6*a*	2*P*_4*A*2*a*_(*t*−1)*P*_6*a*_(*t*−1)	0	0	0	1/5 + 1/5*α*	3/5–1/3*α*	1/5 + 1/15*α*	1/15*α*
3*A*3*a* × 3*A*3*a*	*P*_3*A*3*a*_^2^(*t*−1)	1/400 + 9/400α^2^ + 3/200α	9/200–9/200α^2^ + 3/25α	99/400–9/400α^2^–3/200*α*	41/100 + 9/100α^2^–6/25α	99/400–9/400α^2^–3/200*α*	9/200–9/200α^2^ + 3/25α	1/400 + 9/400α^2^ + 3/200α
3*A*3*a* × 2*A*4*a*	2*P*_3*A*3*a*_(*t*−1)*P*_2*A*4*a*_(*t*−1)	1/100α^2^ + 1/300α	1/100 + 19/300α	3/25–7/100α^2^ + 31/300α	37/100 + 2/25α^2^–59/300α	37/100 + 3/100α^2^–11/75α	3/25–2/25α^2^ + 2/15α	1/100 + 3/100α^2^ + 1/25α
3*A*3*a* × 1*A*5*a*	2*P*_3*A*3*a*_(*t*−1)*P*_1*A*5*a*_(*t*−1)	0	1/40α^2^ + 1/120α	1/40–3/40α^2^ + 2/15α	1/4 + 1/20α^2^–1/15α	9/20 + 1/20α^2^–13/60α	1/4–3/40α^2^ + 7/120α	1/40 + 1/40α^2^ + 1/12α
3*A*3*a* × 6*a*	2*P*_3*A*3*a*_(*t*−1)*P*_6*a*_(*t*−1)	0	0	0	1/20 + 3/20*α*	9/20–3/20*α*	9/20–3/20*α*	1/20 + 3/20*α*
2*A*4*a* × 2*A*4*a*	*P*_2*A*4*a*_^2^(*t*−1)	1/225α^2^	2/225α^2^ + 2/75α	1/25–9/225α^2^ + 8/75α	6/25–4/225α^2^–2/75α	11/25 + 31/225α^2^–22/75α	6/25–2/15α^2^ + 8/75α	1/25 + 1/25α^2^ + 2/25α
2*A*4*a* × 1*A*5*a*	2*P*_2*A*4*a*_(*t*−1)*P*_1*A*5*a*_(*t*−1)	0	1/90α^2^	–1/90α^2^ + 1/15α	1/10–1/15α^2^ + 1/10α	2/5 + 7/45α^2^–4/15α	2/5–11/90α^2^–1/30α	1/10 + 1/30α^2^ + 2/15α
2*A*4*a* × 6*a*	2*P*_2*A*4*a*_(*t*−1)*P*_6*a*_(*t*−1)	0	0	0	1/15*α*	1/5 + 1/15*α*	3/5–1/3*α*	1/5 + 1/5*α*
1*A*5*a* × 1*A*5*a*	*P*_1*A*5*a*_^2^(*t*−1)	0	0	1/36α^2^	–1/9α^2^ + 1/6α	1/4 + 1/6α^2^–1/6α	1/2–1/9α^2^–1/6α	1/4 + 1/36α^2^ + 1/6α
1*A*5*a* × 6*a*	2*P*_1*A*5*a*_(*t*−1)*P*_6*a*_(*t*−1)	0	0	0	0	1/6*α*	1/2–1/3*α*	1/2 + 1/6*α*
6*a* × 6*a*	*P*_6*a*_^2^(*t*−1)	0	0	0	0	0	0	1
Offspring Genotype Frequency		*P*_6*A*_(*t*)	*P*_5*A*1*a*_(*t*)	*P*_4*A*2*a*_(*t*)	*P*_3*A*3*a*_(*t*)	*P*_2*A*4*a*_(*t*)	*P*_1*A*5*a*_(*t*)	*P*_6*a*_(*t*)

**Figure 1:**
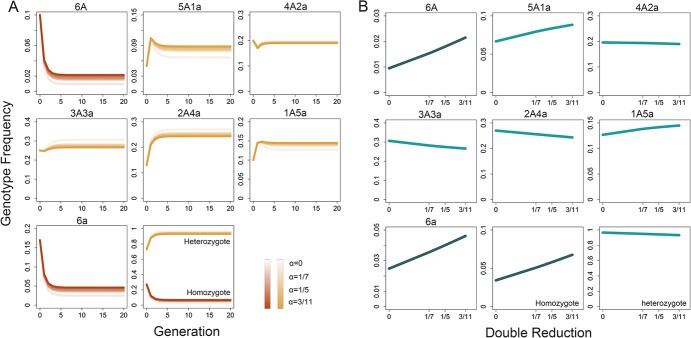
Genotype frequencies at an assumed locus change with generation in a full-sib family of autohexaploid chrysanthemum. (A) Seven genotypes each have a stable frequency after random mating of about 8 generations under different degrees of double reduction. (B) The frequencies of seven genotypes at aHWE change with double reduction.

Table S1 represents a group of recursive equations that describe how genotype frequencies change from one generation to the next in a panmictic hexaploid population. By plotting these frequencies against generation, we can monitor how and when the hexaploid population reaches equilibrium in genotype proportions. Under random chromatid segregation, the rate of double reduction (α) in autohexaploids has a theoretical bound of 0 < α < 3/11 [20]. We randomly sample an array of genotype frequencies P(0) = (0.1, 0.05, 0.2, 0.25, 0.13, 0.1, 0.17) as initial values and plot generation-varying frequencies under α = 0, 1/7, 1/5, 3/11 (Fig. 1A). We find that unlike a case in diploids using one generation to attain HWE, all genotype frequencies in hexaploids will not reach absolute equilibrium but will rather tend to be stable after 8–9 generations of random mating, as opposed to 5–6 generations in tetraploids. Given its asymptotic stability, Sun et al. [17] named such an equilibrium asymptotic HWE (aHWE). We find that double reduction has little impact on the attainment of aHWE in autohexaploids, but it affects the values of equilibrium genotype frequencies (Fig. 1B), suggesting that double reduction is a driver of hexaploid evolution. The above findings are confirmed by repeating our sampling procedure 1000 times.

**How is aHWE tested?** Sun et al. [17] proposed two approaches for testing aHWE in tetraploids. The first is the recursive test based on comparison between the initial genotype frequencies and the genotype frequencies at asymptotic equilibrium. Let P(8) denote an array of genotype frequencies at generation 8 of random mating, estimated by recursive equations (Supplemental Text 1), as aHWE genotype frequencies. By comparing P(8) to the initial (observed) genotype frequencies P(0), we calculate the chi-square test statistic,

(1) \begin{align*} {\chi}^2=N\sum_{j=1}^7\left(\frac{{\left({P}_j(0)-{P}_j(8)\right)}^2}{P_j(8)}\right), \end{align*}

$$ \begin{align*} j=6A,5A1a,4A2a,3A3a,2A4a,1A5a,6a \end{align*}$$

and compare it against the critical threshold ${\chi}_{95\%,\mathrm{df}=6}^2$, from which the significance of deviation from aHWE can be determined.

The second approach is the gamete-based test. Under HWE, genotype frequencies are expressed as the products of gamete frequencies, which are thought to be the expected genotype frequencies. Let *P_AAA_*(t), *P_AAa_*(t), *P_Aaa_*(t), and *P_aaa_*(t) denote the frequencies of four gametes that produce zygotic genotypes at generation *t*, whose equilibrium frequencies are expressed as:

(2) \begin{align*}\begin{aligned} Q_{6A}(t)&=P_{AAA}^{2}(t)\\ Q_{5Aa}(t)&=2P_{AAA}(t)P_{AAa}(t)\\ Q_{4A2a}(t)&=2P_{AAA}(t)P_{Aaa}(t)+P_{AAa}^{2}(t)\\ Q_{3A3a}(t)&=2P_{AAA}(t)P_{aaa}(t)+2P_{AAa}(t)P_{Aaa}(t)\\ Q_{2A4a}(t)&=2P_{AAa}(t)P_{aaa}(t)+P_{Aaa}^{2}(t)\\ Q_{1A5a}(t)&=2P_{Aaa}(t)P_{aaa}(t)\\ Q_{6a}(t)&=P_{aaa}^{2}(t). \end{aligned}\end{align*}

Note that the above expressions of zygote genotype frequencies are derived without considering double reduction because, as shown above (Fig. 1B), its impact on equilibrium frequencies is trivial. Let *N_j_* denote the size of the zygotic genotypes,$6A,5A1a,4A2a,3A3a,2A4a,1A5a,\break\mathrm{and}\ 6a$, observed in the current population. Based on the equilibrium zygotic frequencies in equation (3), we formulate a likelihood of these observations as

(3) \begin{align*} L=\prod_{j=1}^7{N}_j\log \left({Q}_j(t)\right)\end{align*}

where terms related to the heterozygotes $4A2a,3A3a,\mathrm{and}\break 2A4a$ each contain two mixture components. We implemented the expectation–maximization (EM) algorithm to obtain the maximum likelihood estimates (MLEs) of *P_AAA_*(t), *P_AAa_*(t), *P_Aaa_*(t), and *P_aaa_*(t), which are used to estimate the expected frequencies of zygotic genotypes using equation (2) (see also Sun et al. [17]). A chi-square test statistic is calculated to test whether the marker deviates from HWE in hexaploids. Unlike the case of a three-genotype diploid population in which the degree of freedom is equal to 3 − 1 − 1 = 1 [21] for the HWE test, this test statistic follows the chi-square distribution with an unknown degree of freedom. However, we can empirically determine it as a value between 7 − 1 − 1 = 5 to 7 − 1 = 6.

**How can double reduction be tested?** We develop a procedure for testing the significance of double reduction in autohexaploids. Table 2 shows how zygotic genotypes are formed through random mating in the previous generation through a total of 15 mating types. If there is no double reduction (*α* = 0), zygotic frequencies in the current population are reduced from full recursive equations (Supplemental Text 1) as

$$ \begin{align*} {R}_{6\mathrm{A}}={\left({P}_{6A}+\frac{1}{2}{P}_{5A1a}+\frac{1}{5}{P}_{4A2a}+\frac{1}{20}{P}_{3A3a}\right)}^2 \end{align*}$$

$$ \begin{align*} {R}_{5\mathrm{A}1\mathrm{a}} &=\frac{1}{200}(20{P}_{6A}+10{P}_{5A1a}+4{P}_{4A2a}+{P}_{3A3a})(10{P}_{5A1a}\\
&\quad +12{P}_{4A2a}+9{P}_{3A3a}+4{P}_{2A4a}) \end{align*}$$

$$ \begin{align*} {R}_{4\mathrm{A}2\mathrm{a}}&=\frac{1}{400}\big(100{P}_{5A1a}^2+176{P}_{4A2a}^2+99{P}_{3A3a}^2+16{P}_{2A4a}^2\\
&\quad+160{P}_{6A}{P}_{4A2a}+360{P}_{6A}{P}_{3A3a}+480{P}_{6A}{P}_{2A4a}\\&\quad
+400{P}_{6A}{P}_{1A5a}+320{P}_{5A1a}{P}_{4A2a}+360{P}_{5A1a}{P}_{3A3a}\\
&\quad +320{P}_{5A1a}{P}_{2A4a}+200{P}_{5A1a}{P}_{1A5a}+296{P}_{4A2a}{P}_{3A3a}\\
&\quad
+192{P}_{4A2a}{P}_{2A4a}+80{P}_{4A2a}{P}_{1A5a}+96{P}_{3A3a}{P}_{2A4a}\\
&\quad +20{P}_{3A3a}{P}_{1A5a}\big) \end{align*}$$

(4) \begin{align*}\notag {R}_{3\mathrm{A}3\mathrm{a}}&=\frac{1}{100}\big(24{P}_{4A2a}^2+41{P}_{3A3a}^2+24{P}_{2A4a}^2+100{P}_{6A}{P}_{3A3a}\\[.2ex] &\quad\notag+40{P}_{6A}{P}_{2A4a}+100{P}_{6A}{P}_{1A5a}+200{P}_{6A}{P}_{6a}\\[.2ex] &\quad\notag +20{P}_{5A1a}{P}_{4A2a}+50{P}_{5A1a}{\mathrm{P}}_{3A3a}+80{P}_{5A1a}{P}_{2A4a}\\&\quad\notag +100{P}_{5A1a}{P}_{1A5a}+100{P}_{5A1a}{P}_{6a}+74{P}_{4A2a}{P}_{3A3a}\\&\quad\notag +88{P}_{4A2a}{P}_{2A4a}+80{P}_{4A2a}{P}_{1A5a}+40{P}_{4A2a}{P}_{6a}\\[.2ex] &\quad\notag +74{P}_{3A3a}{P}_{2A4a}+50{P}_{3A3a}{P}_{1A5a}+10{P}_{3A3a}{P}_{6a}\\[.2ex]&\quad +20{P}_{2A4a}{P}_{1A5a}\big) \end{align*}

$$ \begin{align*} {R}_{2\mathrm{A}4\mathrm{a}}&=\frac{1}{400}\big(16{P}_{4A2a}^2+99{P}_{3A3a}^2+176{\mathrm{P}}_{2A4a}^2+100{P}_{1A5a}^2\\
&\quad+20{P}_{5A1a}{P}_{3A3a}+80{P}_{5A1a}{P}_{2A4a}+200{P}_{5A1a}{P}_{1A5a}\\
&\quad+400{P}_{5A1a}{P}_{6a}+96{P}_{4A2a}{P}_{3A3a}+192{P}_{4A2a}{P}_{2A4a}\\
&\quad+320{P}_{4A2a}{P}_{1A5a}+480{P}_{4A2a}{P}_{6a}+296{P}_{3A3a}{P}_{2A4a}\\
&\quad+360{P}_{3A3a}{P}_{1A5a}+360{P}_{3A3a}{P}_{6a}+320{P}_{2A4a}{P}_{1A5a}\\&\quad
+160{P}_{2A4a}{P}_{6a}\big) \end{align*}$$

$$ \begin{align*} {R}_{5\mathrm{A}1\mathrm{a}}&=\frac{1}{200}\big(4{P}_{4A2a}+9{P}_{3A3a}+12{P}_{2A4a}+10{P}_{1A5a}\big)\big({P}_{3A3a}\\
&\quad+4{P}_{2A4a}+10{P}_{1A5a}+20{P}_{6a}\big) \end{align*}$$

$$ {R}_{6\mathrm{a}}={\left(\frac{1}{20}{P}_{3A3a}+\frac{1}{5}{P}_{2A4a}+\frac{1}{2}{P}_{1A5a}+{P}_{6a}\right)}^2 $$

where *P_j_*’s ($j=6A,5A1a,4A2a,3A3a,2A4a,1A5a,6a$) are the zygotic frequencies in the parental population.

We formulate a likelihood of observations of seven zygotic genotypes based on the zygotic frequencies of equation (4) under α = 0, which is expressed as

(5) \begin{align*} L=\prod_{j=1}^7{N}_j\log \left({R}_j\right)\end{align*}

where each term contains complex mixture components. We take advantage of the EM algorithm described in Sun et al. [17] to estimate the genotype frequencies *P_j_*’s in the parental population. In Supplemental Text 2, we provide a detailed procedure for the EM algorithm for genotype frequency estimation. By substituting the MLEs of these parental frequencies into equation (4), we obtain the MLEs of zygotic frequencies *R_j_*’s in the current generation under the assumption of no double reduction. Thus, by comparing the observations of genotypes, we use a chi-square test to determine whether double reduction exists at the considered SNP by calculating the test statistic

(6) \begin{align*} {\chi}^2=N\sum_{j=1}^7\frac{{\left({P}_j-{R}_j\right)}^2}{R_j}\end{align*}

which is χ^2^-distributed with five to six degrees of freedom.

Next, we describe a procedure for estimating the MLE of double reduction. Because double reduction has little influence on equilibrium genotypic frequencies, we can replace genotype frequencies at generation *t* − 1 contained in recursive equations (Supplemental Text 1) by equilibrium genotype frequencies expressed as the products of maternal gamete frequencies and paternal gamete frequencies, with a similar form of equation (2). Thus, recursive equations are composed of gamete frequencies ${P}_{AAA}(t-1)$, ${P}_{AAa}(t-1)$, ${P}_{Aaa}(t-1)$ and ${P}_{aaa}(t-1)$ and the rate of double reduction *α*. Similar to equation (3), we formulate a likelihood based on seven genotype frequencies at generation *t*. Each term in the likelihood contains complex mixture components. Thus, we implement the EM algorithm to estimate ${P}_{AAA}(t-1)$, ${P}_{AAa}(t-1)$, ${P}_{Aaa}(t-1)$, ${P}_{aaa}(t-1)$, and *α*.

The by-product of the above procedure is to test whether the parent population at generation *t* – 1 deviates from aHWE. Under *α* ≠ 0, we calculate the likelihood (L_1_) from equation (5), which corresponds to the alternative hypothesis that there is deviation from aHWE. Meanwhile, we calculate the likelihood (L_0_) of the case in which parental genotype frequencies are expressed as equilibrium frequencies, which corresponds to the null hypothesis. The log-likelihood ratio

(7) \begin{align*} \text{LR}=-2(\text{log}\ \text{L}_{0}-\text{log}\ \text{L}_{1}) \end{align*}

is a test statistic assumed to follow a chi-square distribution with one degree of freedom. This procedure can test the existence of parental aHWE.

## Numerical examples

**Test procedure:** As an example that demonstrates how to test aHWE and double reduction, we generate a random set of seven hexaploid genotypes containing 29 individuals for 6*A*, 21 for 5*A*1*a*, 17 for 4*A*2*a*, 10 for 3*A*3*a*, 10 for 2*A*4*a*, 10 for 1*A*5*a*, and 23 for 6*a* at a SNP, totaling *N* = 120, from a natural autohexaploid population. We use recursive equations (Supplemental Text 1) to estimate aHWE genotype frequencies at generation 8 of random mating. By comparing these equilibrium frequencies with observed genotype frequencies, we calculate a chi-square test statistic, which is 6.602 (compared with ${\chi}_{95\%,\mathrm{df}=6}^2$), suggesting that the segregation of the marker deviates from HWE. We implement the EM algorithm (Supplemental Text 2) to estimate gamete frequencies under HWE and use these estimates to obtain the MLEs of equilibrium zygotic frequencies. The chi-square test statistic is calculated as 6.649, indicating significant deviation from HWE. Thus, both the recursive test and the gamete-based test produce a consistent result in this example.

To test whether this example contains significant double reduction, we implement the EM algorithm to estimate zygotic frequencies (under the assumption of no double reduction) in the parental generation and use these estimates to obtain the expected zygotic frequencies in the current generation. Then, using equation (6), we calculate the chi-square test statistic as 5.922, which suggests the existence of double reduction in this example.

**Monte Carlo simulation:** We performed computer simulation to examine the statistical properties of our EM algorithm-based testing procedure. Simulation studies focus on assessing the estimation precision of parental gamete frequencies and parental zygotic genotype frequencies, which are used to test the existence of aHWE and double reduction. We sample a set of parental genotype frequencies P(*t*−1) = (*P*_6A_(*t*−1), *P*_5A1a_(*t*−1), *P*_4A2a_(*t*−1), *P*_3A3a_(*t*−1), *P*_2A4a_(*t*−1), *P*_1A5a_(*t*−1), *P*_6a_(*t*−1)) = (0.10, 0.5, 0.20, 0.25, 0.13, 0.10, 0.17) under sample sizes of *N* = 100, 200, and 400. Under different values of *α*, we use recursive equations (Supplemental Text 1) to estimate offspring genotype frequencies. By assuming parental aHWE, we use the EM algorithm to estimate parental gamete frequencies (*P_AAA_*(*t*−1), *P_AAa_*(*t*−1), *P_Aaa_*(*t*−1), *P_aaa_*(*t*−1)) and *α*. As shown in Table 3, all these parameters can be estimated with reasonable precision, even with a modest sample size of *N* = 100. The power of detecting aHWE is about 0.70 for *N* = 100 to about 0.90 for *N* = 400. We simulate another set of offspring genotype frequencies from parental gamete frequencies under aHWE from which to estimate the probability of incorrectly detecting aHWE. Such false positive rates are quite low, below 0.08, under different sample sizes.

**Table 3:** MLEs of gamete frequencies and their standard deviations and the empirical power of the aHWE test estimated from simulated data for a natural panmictic autohexaploid population under different degrees of double reduction and sample sizes

Estimates of parental genotype frequencies													
		*α* = 0			1/7			1/5			1/5		
Gamete	True Value	*n* = 100	200	400	100	200	400	100	200	400	100	200	400
*AAA*	*P_AAA_* = 0.30	0.288 ± 0.026	0.289 ± 0.051	0.293 ± 0.036	0.313 ± 0.104	0.310 ± 0.050	0.311 ± 0.025	0.327 ± 0.033	0.319 ± 0.029	0.314 ± 0.025	0.356 ± 0.035	0.339 ± 0.030	0.340 ± 0.028
*AAa*	*P_AAa_* = 0.20	0.231 ± 0.055	0.228 ± 0.043	0.220 ± 0.033	0.222 ± 0.073	0.207 ± 0.068	0.201 ± 0.064	0.209 ± 0.070	0.203 ± 0.066	0.200 ± 0.060	0.179 ± 0.063	0.192 ± 0.061	0.194 ± 0.054
*Aaa*	*P_Aaa_* = 0.35	0.318 ± 0.138	0.321 ± 0.131	0.334 ± 0.079	0.337 ± 0.077	0.340 ± 0.074	0.343 ± 0.070	0.333 ± 0.108	0.323 ± 0.074	0.331 ± 0.069	0.322 ± 0.077	0.316 ± 0.076	0.319 ± 0.068
*aaa*	*P_aaa_* = 0.15	0.163 ± 0.066	0.162 ± 0.039	0.159 ± 0.034	0.130 ± 0.059	0.143 ± 0.032	0.145 ± 0.030	0.134 ± 0.063	0.155 ± 0.035	0.157 ± 0.033	0.143 ± 0.043	0.153 ± 0.039	0.147 ± 0.037
*α*		0.007 ± 0.089	0.007 ± 0.077	0.005 ± 0.065	0.127 ± 0.139	0.127 ± 0.107	0.131 ± 0.089	0.172 ± 0.125	0.176 ± 0.099	0.177 ± 0.111	0.224 ± 0.149	0.225 ± 0.132	0.232 ± 0.093

Correctly testing for the existence of double reduction depends on the precise estimation of parental genotype frequencies based on the EM algorithm. We examine the precision and power of parameter estimation through computer simulation studies. Given initial values for parental genotype frequencies P(*t*−1) = (0.10, 0.05, 0.20, 0.25, 0.13, 0.10, 0.17), we simulate the observations of seven genotypes in the current population under *α* = 0, 1/7, 1/6, 1/5, 1/4, assuming sample size *N* = 100, 200, and 400. The means and standard deviations of the estimates of each parental genotype frequency and the power for detecting significant double reduction are given in Table 4. It can be seen that parental genotype frequencies can be fairly well estimated even under a modest sample size (*N* = 100), although the accuracy and precision of parameter estimates increase with sample size. Considering the adequate power of detecting double reduction, a sample size of at least *N* = 100 is recommended to obtain reasonably good estimates of parental genotype frequencies and, therefore, a good test for the occurrence of double reduction in an autohexaploid natural population. If the signal of double reduction is weak, more samples (say *N* > 200) are needed to reasonably detect double reduction. If double reduction is detected for the simulated offspring zygote frequency data under no double reduction, then this indicate a false positive discovery. We find that our model has reasonably low false positive rates (<0.10) even under a small sample size.

**Table 4:** MLEs of parental zygote frequencies and their standard deviations and the empirical power of double reduction detection estimated from simulated data for a natural panmictic autohexaploid population under different degrees of double reduction and sample sizes

Estimates of Parental Genotype Frequencies																
		α = 0			1/7			1/6			1/5			1/4		
Genotype True Value		n = 100	200	400	100	200	400	100	200	400	100	200	400	100	200	400
6*A*	P_6_ = 0.10	0.102 ± 0.065	0.103 ± 0.051	0.101 ± 0.035	0.1116 ± 0.067	0.116 ± 0.53	0.117 ± 0.040	0.118 ± 0.070	0.116 ± 0.052	0.118 ± 0.038	0.120 ± 0.070	0.121 ± 0.053	0.122 ± 0.041	0.126 ± 0.071	0.126 ± 0.053	0.127 ± 0.044
5*A*1*a*	P_5_ = 0.05	0.046 ± 0.029	0.049 ± 0.023	0.049 ± 0.015	0.049 ± 0.030	0.052 ± 0.022	0.052 ± 0.016	0.052 ± 0.032	0.052 ± 0.022	0.052 ± 0.016	0.052 ± 0.032	0.051 ± 0.022	0.052 ± 0.016	0.052 ± 0.029	0.053 ± 0.023	0.053 ± 0.016
4*A*2*a*	P_4_ = 0.20	0.210 ± 0.124	0.204 ± 0.092	0.199 ± 0.061	0.200 ± 0.114	0.197 ± 0.085	0.192 ± 0.059	0.200 ± 0.114	0.197 ± 0.084	0.192 ± 0.057	0.200 ± 0.115	0.191 ± 0.082	0.190 ± 0.057	0.196 ± 0.106	0.190 ± 0.077	0.186 ± 0.056
3*A*3*a*	P_3_ = 0.25	0.228 ± 0.066	0.233 ± 0.039	0.244 ± 0.024	0.212 ± 0.067	0.218 ± 0.046	0.226 ± 0.032	0.205 ± 0.071	0.218 ± 0.045	0.224 ± 0.033	0.202 ± 0.072	0.216 ± 0.047	0.220 ± 0.037	0.201 ± 0.071	0.209 ± 0.051	0.214 ± 0.041
2*A*4*a*	P_2_ = 0.13	0.151 ± 0.115	0.139 ± 0.072	0.137 ± 0.048	0.141 ± 0.099	0.127 ± 0.063	0.127 ± 0.043	0.136 ± 0.095	0.127 ± 0.060	0.126 ± 0.042	0.133 ± 0.088	0.129 ± 0.059	0.123 ± 0.042	0.125 ± 0.078	0.123 ± 0.057	0.121 ± 0.040
1*A*5*a*	P_1_ = 0.10	0.103 ± 0.064	0.106 ± 0.053	0.105 ± 0.037	0.108 ± 0.069	0.105 ± 0.053	0.103 ± 0.036	0.108 ± 0.071	0.104 ± 0.051	0.103 ± 0.036	0.109 ± 0.071	0.107 ± 0.051	0.103 ± 0.036	0.106 ± 0.065	0.105 ± 0.050	0.103 ± 0.035
6*a*	P_0_ = 0.17	0.161 ± 0.082	0.167 ± 0.062	0.165 ± 0.045	0.175 ± 0.079	0.183 ± 0.061	0.183 ± 0.044	0.181 ± 0.089	0.185 ± 0.060	0.185 ± 0.045	0.184 ± 0.080	0.185 ± 0.060	0.189 ± 0.045	0.194 ± 0.079	0.193 ± 0.061	0.196 ± 0.048
Rejecting null hypothesis		0.092	0.086	0.083	0.667	0.713	0.750	0.708	0.717	0.762	0.709	0.727	0.779	0.742	0.785	0.855

The proportion of rejecting the null hypothesis is the power of double reduction detection for the simulated data under *α* ≠ 0 and the rate of false positive discovery under *α* = 0.

**Real data analysis:** To demonstrate how our methods can be used to test for aHWE and double reduction, we analyze marker data collected from an autohexaploid chrysanthemum with great ornamental and medicinal value [22]. As an allogamous plant, chrysanthemum has six sets of chromosomes, each with 9 chromosomes, and its numerous chromosomes (2n = 6x = 54) make it difficult to study the genome structure of this species without sophisticated statistical methods. By crossing two heterozygous parents, Sumitomo et al. [22] generated a segregating full-sib family, which can be used as a proxy for a natural population in terms of the pattern of marker segregation. For this family, a total of 5509 intercross simplex markers and 3710 testcross simplex markers were genotyped. Yet, because a low-resolution sequencing technique was used, these markers are dosage ambiguous, i.e. it is impossible to distinguish the five heterozygotes 5*A*1*a*, 4*A*2*a*, 3*A*3*a*, 2*A*4*a*, and 1*A*5*a* (collectively denoted *A*_*a*_) from one another. We randomly choose four segregating markers for equilibrium and double reduction tests.

Table 5 presents the result of marker tests. By comparing observed genotype frequencies with equilibrium genotype frequencies calculated at generation 8 after random mating, the recursive test finds that all chosen markers significantly deviate from aHWE, and the two markers SNP-113 and SNP-312 have p-values of <10^−50^. Because there are only three distinguishable genotypes, a gamete-based approach cannot be used to test for aHWE. To do so, we implement an allele-based approach, assuming that the formation of a triploid gamete involves the random combination of three alleles, i.e. the frequencies of *AAA*, *AAa*, *Aaa*, and *aaa* are expressed as *p*^3^, 2*p*^2^*q*, 2*pq*^2^, and *q*^3^, where *p* and *q* are the allele frequencies of *A* and *a*, respectively. A chi-square test based on the allele model produces equilibrium test results that are highly consistent with those obtained from the recursive test (Table 5).

**Table 5:** Examples of the aHWE test at four randomly chosen SNPs in a full-sib family of autohexaploid chrysanthemum

SNP ID		SNP-4			SNP-18			SNP-113			SNP-312		
Genotype		6*A*	*A*_*a*_	6*a*	6*A*	*A*_*a*_	6*a*	6*A*	*A*_*a*_	6*a*	6*A*	*A*_*a*_	6*a*
Recursive	Observed frequency	0.4000	0.4875	0.1125	0.5753	0.3699	0.0548	0.7826	0.1884	0.0290	0.8254	0.1587	0.0159
	Expected frequency	0.0714	0.9266	0.0020	0.1934	0.8064	0.0002	0.4546	0.5454	3.48 × 10^−6^	0.5484	0.4516	7.50 × 10^−7^
	Chi-square value	7.63			16.44			231.14			320.65		
	*p*-value	0.0220			0.0003			6.42 × 10^−51^			2.35 × 10^−70^		
Allele-based	Observed frequency	0.4000	0.4875	0.1125	0.5753	0.3699	0.0548	0.7826	0.1884	0.0290	0.8254	0.1587	0.0159
	Expected frequency	0.0712	0.9268	0.0020	0.1935	0.8063	0.0002	0.4548	0.5452	3.64 × 10^−6^	0.5489	0.4511	7.86 × 10^−7^
	Chi-square value	7.69			16.80			241.39			337.17		
	*p*-value	0.0214			0.0002			3.82 × 10^−53^			6.09 × 10^−74^		

By incorporating the allele model into recursive equations (Supplemental Text 1), we can test the significance of double reduction at individual heterozygote-ambiguous markers. Table 6 illustrates such test results at four randomly chosen markers. We find that marker SNP-5 does not display significant double reduction, whereas double reduction is highly significant at markers SNP-130, SNP-406, and SNP-558. Our model provides a unique tool for testing double reduction.

**Table 6:** Examples of testing double reduction at four randomly chosen SNPs in a full-sib family of autohexaploid chrysanthemum

SNP ID	SNP-5			SNP-130			SNP-406			SNP-558		
Genotype	6*A*	*A*_*a*_	6*a*	6*A*	*A*_*a*_	6*a*	6*A*	*A*_*a*_	6*a*	6*A*	*A*_*a*_	6*a*
Observed frequency	0.1539	0.6593	0.1868	0.6308	0.3385	0.0308	0.5303	0.3030	0.1667	0.0421	0.3053	0.6526
Expected frequency	0.0099	0.9662	0.0239	0.5186	0.4814	1.24 × 10^−06^	0.1628	0.0369	0.0003	1.64 × 10^−06^	0.4984	0.5016
Chi-square value	3.31			763.6			88.5			1078.39		
*p*-value	0.19			1.53 × 10^−166^			5.94 × 10^−20^			6.76 × 10^−235^		

## Discussion

Polyploids are a group of plants with great importance in plant evolutionary studies and plant breeding. Although there is a rich body of literature on quantitative genetic dissection of complex traits based on artificial crosses [23,24,25], only a few studies have investigated the population genetic diversity of polyploids [4,15,16,26,27]. There are few methodological studies that describe analytical models for population and evolutionary genetics in polyploids by considering the structural and organizational complexities of polyploid genomes [28]. Sun et al. [17] developed a simple mathematical model to confirm the number of generations required to asymptotically approach HWE in tetraploids by early geneticists [18], but beyond this detection, Sun et al. proposed a statistical procedure for testing aHWE and validated its usefulness by analyzing a real data set. It can be expected that the conclusion of Sun et al. can be extended to polyploids at a higher ploidy level, but a convincing proof and the corresponding algorithm for the equilibrium test are not available.

In this article, we propose a mathematical procedure for detecting equilibrium genotype frequencies in a panmictic hexaploid population by deriving a group of recursive equations. We find that in contrast to diploid populations that reach HWE after only one generation of random mating, hexaploids require at least eight generations to approach asymptotic equilibrium. This is also different from tetraploids, which require four generations of random mating [17]. These recursive equations provide a general framework for testing aHWE from different perspectives. A so-called recursive test attempts to compare observed genotype frequencies with equilibrium genotype frequencies calculated at generation 8 after random mating. Using the standard equilibrium assumption, we develop a statistical gamete-based algorithm for HWE testing in parental and offspring hexaploid populations. As seen from several numerical examples, both recursive and statistical methods produce consistent test results.

One additional advantage of our procedure is the ability to estimate and test double reduction in autohexaploids. As a common phenomenon with a role in shaping autopolyploid diversity and evolution, double reduction has received considerable attention [19,29]. Yet, its estimation and testing are mostly performed using artificial controlled crosses [30], although a few studies have done so using a panel of samples from natural populations [31]. In this study, we incorporate recursive equations to test the significance of double reduction over the autohexaploid genome. This procedure can scan molecular markers throughout the genome, visualize the landscape of double reduction, and identify key regions where this phenomenon occurs. In many polyploid genetic studies, genome sequencing is not conducted at a level of high resolution that allows heterozygous genotypes to be distinguished from each other in terms of allelic dosages. For these genotype-ambiguous markers, we incorporate an allele-based model to test double reduction by assuming that gametes are random combinations of paternal and maternal alleles. This allele-based model expands the application of our test procedure to test double reduction using less informative markers.

We perform computer simulation to examine the statistical properties of our procedure, validating its usefulness. To test aHWE in hexaploid populations, a modest sample size (say 100) is adequate for the recursive approach because it only relies on the estimation of seven genotype frequencies. The gamete-based approach requires a reasonable estimate of gamete frequencies by the EM algorithm, which requires a larger sample size (say 200) for the aHWE test. In addition, results from computer simulation suggest that a modest sample size of 100 can reasonably estimate the genotypic frequencies, with good power to detect the significance of a small double reduction (*α* = 1/7). A low false positive rate implies that as long as double reduction is tested to be significant, the likelihood of its actual existence is high. As a proof of concept, we use our procedure to analyze data from a full-sib family of autohexaploid chrysanthemum [21]. Although these data were not collected from a natural population, marker segregation in the family follows a similar pattern to that expected in nature. Thus, it is reasonable to demonstrate the utility of our procedure using a full-family dataset. We show our test results by randomly choosing several markers (Tables 4 and 5) and further explain these results in terms of aHWE and double reduction by our procedure.

In conclusion, our computational procedure is robust for testing aHWE and the occurrence of double reduction. It could have immediate implications for analyzing population genetic data collected from natural populations of any autohexaploid or allohexaploid species, including sweetpotato, wheat, kiwifruit, etc. Results from our procedure can provide insight into the evolutionary forces that act on the genomes of hexaploids and can also be used to detect genotyping errors in marker data. As the first step of molecular breeding in hexaploids, genome-wide association studies (GWAS) have been increasingly used as a routine approach for studying the genetic architecture of agriculturally important traits [32–34]. Our aHWE testing procedure provides valuable assistance for the quality control of markers and the evolutionary inference of any significant loci detected from GWAS. In this study, we focus our analysis and modeling on single markers, but a joint analysis of two, even more than two, markers is essential, despite its tediousness in model derivations, given that non-random associations between different markers [28] (as modeled in tetraploids) contribute to hexaploid diversity and evolution in a different way.

## Acknowledgments

We thank Dr. Libo Jiang for his contribution to this work and Beijing Forestry University for providing funds to support this project. This work was supported by the Forestry and Grassland Science and Technology Innovation Youth Top Talent Project of China (No. 2020132608), the National Natural Science Foundation of China (No. 31870689), and the National Key Research and Development Program of China (2018YFD1000401).

## Author Contributions

JW derived the model, analyzed the data, and developed the code. LF, SM, AD, JG, ZW, JM, and ML participated in model derivation and theme discussion. LS conceived the study and supervised the project. LS and RW drafted the manuscript with inputs from all other authors.

## Data availability

All the data and code are deposited and may be freely downloaded at https://github.com/CCBBeijing/hexaploid/. They may also be requested from the corresponding author.

## Conflict of interest

The authors declare no competing interests.

## Supplementary data

Supplementary data is available at *Horticulture Research * online.

## Supplementary Material

suppl_uhac104
