# Supplementary material for: Asymptotic tests for Hardy–Weinberg equilibrium in hexaploids
Source: Hortic Res. 2022 May 17;9:uhac104. doi: 10.1093/hr/uhac104 (PMC9250657; doi:10.1093/hr/uhac104)
Supplement: suppl_uhac104 [file suppl_uhac104.zip › Hexaploid_sup.docx]

**Supplemental Text 1:** Zygote genotype frequencies in the offspring population derived from 15 types of parental mating as given in Table 2.

P_6A_(t+1) =

P_6A_^2^(t)+2P_6A_(t)P_5A1a_(t)(1/2+1/6α)+2P_6A_(t)P_4A2a_(t)(1/5+1/5α)+2P_6A_(t)P_3A3a_(t)(1/20+3/20α)

+2P_6A_(t)P_2A4a_(t)(1/15α)+P_5A1a_^2^(t)(1/4+1/36α^2^+1/6α)+2P_5A1a_(t)P_4A2a_(t)(1/10+1/30α^2^+2/15α)

+2P_5A1a_(t)P_3A3a_(t)(1/40+1/40α^2^+1/12α)+2P_5A1a_(t)P_2A4a_(t)(1/90α^2^+1/30α)+P_4A2a_^2^(t)(1/25+1/25α^2^

+2/25α)+2P_4A2a_(t)P_3A3a_(t)(1/100+3/100α^2^+1/25α)+2P_4A2a_(t)P_2A4a_(t)(1/75α^2^+1/75α) +P_3A3a_^2^(t)(1/400+9/400α^2^+3/200α)+2P_3A3a_(t)P_2A4a_(t)(1/100α^2^+1/300α)+P_2A4a_^2^(t)(1/225α^2^)

P_5A1a_(t+1) =

2P_6A_P_5A1a_(t) (1/2-1/3α)+2P_6A_(t)P_4A2a_(t)(3/5-1/3α)+2P_6A_(t)P_3A3a_(t)(9/20-3/20α)

+2P_6A_(t)P_2A4a_(t)(1/5+1/15α)+2P_6A_(t)P_1A5a_(t)(1/6α)+P_5A1a_^2^(t)(1/2-1/9α^2^-1/6α)

+2P_5A1a_(t)P_4A2a_(t)(2/5-11/90α^2^-1/30α)+2P_5A1a_(t)P_3A3a_(t)(1/4-3/40α^2^+7/120α)

+2P_5A1a_(t)P_2A4a_(t)(1/10-1/90α^2^+1/10α)+2P_5A1a_(t)P_1A5a_(t)(1/36α^2^+1/12α)

+P_4A2a_^2^(t)(6/25-2/15α^2^+8/75α)+2P_4A2a_(t)P_3A3a_(t)(3/25-2/25α^2^+2/15α)

+2P_4A2a_(t)P_2A4a_(t)(1/25-2/225α^2^+7/75α)+2P_4A2a_(t)P_1A5a_(t)(1/30α^2^+1/30α)

+P_3A3a_^2^(t)(9/200-9/200α^2^+3/25α)+2P_3A3a_(t)P_2A4a_(t)(1/100+19/300α)

+2P_3A3a_(t)P_1A5a_(t)(1/40α^2^+1/120α)+P_2A4a_^2^(t)(2/225α^2^+2/75α)+2P_2A4a_(t)P_1A5a_(t)(1/90α^2^)

P_4A2a_(t+1) =

2P_6A_(t)P_5A1a_(t)(1/6α)+2P_6A_(t)P_4A2a_(t)(1/5+1/15α)+2P_6A_(t)P_3A3a_(t)(9/20-3/20α)

+2P_6A_(t)P_2A4a_(t)(3/5-1/3α)+2P_6A_(t)P_1A5a_(t)(1/2-1/3α)+P_5A1a_^2^(t)(1/4+1/6α^2^-1/6α)

+2P_5A1a_(t)P_4A2a_(t)(2/5+7/45α^2^-4/15α)+2P_5A1a_(t)P_3A3a_(t)(9/20+1/20α^2^-13/60α)

+2P_5A1a_(t)P_2A4a_(t)(2/5-1/15α^2^-1/10α)+2P_5A1a_(t)P_1A5a_(t)(1/4-1/9α^2^)+P_4A2a_^2^(t)(11/25+31/225α^2^-22/75α)

+2P_4A2a_(t)P_3A3a_(t)(37/100+3/100α^2^-11/75α)+2P_4A2a_(t)P_2A4a_(t)(6/25-19/225α^2^+1/25α)

+ 2P_4A2a_(t)P_1A5a_(t)(1/10-11/90α^2^+2/15α)+P_3A3a_^2^(t)(99/400-9/400α^2^-3/200α)

+2P_3A3a_(t)P_2A4a_(t)(3/25-7/100α^2^+31/300α)+2P_3A3a_(t)P_1A5a_(t)(1/40-3/40α^2^+2/15α)

+P_2A4a_^2^(t)(1/25-9/225α^2^+8/75α)+2P_2A4a_(t)P_1A5a_(t)((-1/90)α^2^+1/15α)+P_1A5a_^2^(t) (1/36α^2^)

P_3A3a_(t+1) =

2P_6A_(t)P_4A2a_(t)(1/15α)+2P_6A_(t)P_3A3a_(t)(1/20+3/20α)+2P_6A_(t)P_2A4a_(t)(1/5+1/5α)

+2P_6A_(t)P_1A5a_(t)(1/2+1/6α)+2P_6A_(t)P_6a_(t)1+P_5A1a_^2^(t)((-1/9)α^2^+1/6α)

+2P_5A1a_(t)P_4A2a_(t)(1/10-1/15α^2^+1/10α)+2P_5A1a_(t)P_3A3a_(t)(1/4+1/20α^2^-1/15α)

+2P_5A1a_(t)P_2A4a_(t)(2/5+7/45α^2^-1/5α)+2P_5A1a_(t)P_1A5a_(t)(1/2+1/6α^2^-1/6α)+2P_5A1a_(t)P_6a_(t)(1/2+1/6α)

+P_4A2a_^2^(t)(6/25-4/225α^2^-2/75α)+2P_4A2a_(t)P_3A3a_(t)(37/100+2/25α^2^-59/300α)

+2P_4A2a_(t)P_2A4a_(t)(11/25+4/25α^2^-22/75α)+2P_4A2a_(t)P_1A5a_(t)(2/5+7/45α^2^-1/5α)

+2P_4A2a_(t)P_6a_(t)(1/5+1/5α)+P_3A3a_^2^(t)(41/100+9/100α^2^-6/25α)

+2P_3A3a_(t)P_2A4a_(t)(37/100+2/25α^2^-59/300α)+2P_3A3a_(t)P_1A5a_(t)(1/4+1/20α^2^-1/15α)

+2P_3A3a_(t)P_6a_(t)(1/20+3/20α)+P_2A4a_^2^(t)(6/25-4/225α^2^-2/75α)+2P_2A4a_(t)P_1A5a_(t)(1/10-1/15α^2^+1/10α)

+2P_2A4a_(t)P_6a_(t)(1/15α)+P_1A5a_^2^(t)((-1/9)α^2^+1/6α)

P_2A4a_(t+1) =

P_5A1a_^2^(t)(1/36α^2^)+2P_5A1a_(t)P_4A2a_(t)((-1/90)α^2^+1/15α)+2P_5A1a_(t)P_3A3a_(t)(1/40-3/40α^2^+2/15α)

+2P_5A1a_(t)P_2A4a_(t)(1/10-11/90α^2^+2/15α)+2P_5A1a_(t)P_1A5a_(t)(1/4-1/9α^2^)+2P_5A1a_(t)P_6a_(t)(1/2-1/3α)

+P_4A2a_^2^(t)(1/25-9/225α^2^+8/75α)+2P_4A2a_(t)P_3A3a_(t)(3/25-7/100α^2^+31/300α)

+2P_4A2a_(t)P_2A4a_(t)(6/25-19/225α^2^+1/25α)+2P_4A2a_(t)P_1A5a_(t)(2/5-1/15α^2^-1/10α)

+2P_4A2a_(t)P_6a_(t)(3/5-1/3α)+P_3A3a_^2^(t)(99/400-9/400α^2^-3/200α)

+2P_3A3a_(t)P_2A4a_(t)(37/100+3/100α^2^-11/75α)+2P_3A3a_(t)P_1A5a_(t)(9/20+1/20α^2^-13/60α)

+2P_3A3a_(t)P_6a_(t)(9/20-3/20α)+P_2A4a_^2^(t)(11/25+31/225α^2^-22/75α)+2P_2A4a_(t)P_1A5a_(t)(2/5+7/45α^2^-4/15α)

+2P_2A4a_(t)P_6a_(t)(1/5+1/15α)+P_1A5a_^2^(t)(1/4+1/6α^2^-1/6α)+2P_1A5a_(t)P_6a_(t)(1/6α)

P_1A5a_(t+1) =

2P_5A1a_(t)P_4A2a_(t)(1/90α^2^)+2P_5A1a_(t)P_3A3a_(t)(1/40α^2^+1/120α)+2P_5A1a_(t)P_2A4a_(t)(1/30α^2^+1/30α)

+2P_5A1a_(t)P_1A5a_(t)(1/36α^2^+1/12α)+2P_5A1a_(t)P_6a_(t)(1/6α)+P_4A2a_^2^(t)(2/225 α^2^+2/75α)

+2P_4A2a_(t)P_3A3a_(t)(1/100+19/300α)+2P_4A2a_(t)P_2A4a_(t)(1/25-/225α^2^+7/75α)

+2P_4A2a_(t)P_1A5a_(t) (1/10-1/90α^2^+1/10α)+2P_4A2a_(t)P_6a_(t)(1/5+1/15α)+P_3A3a_^2^(t)(9/200-9/200α^2^+3/25α)

+2P_3A3a_(t) P_2A4a_(t)(3/25-2/25α^2^+2/15α)+2P_3A3a_(t)P_1A5a_(t)(1/4-3/40α^2^+7/120α)

+2P_3A3a_(t)P_6a_(t)(9/20-3/20α)+P_2A4a_^2^(t)(6/25-2/15α^2^+8/75α)+2P_2A4a_(t)P_1A5a_(t)(2/5-11/90α^2^-1/30α)

+2P_2A4a_(t)P_6a_(t)(3/5-1/3α)+P_1A5a_^2^(t)(1/2-1/9α^2^-1/6α)+2P_1A5a_(t)P_6a_(t)(1/2-1/3α)

P_6a_(t+1) =

P_4A2a_^2^(t)(1/225α^2^)+2P_4A2a_(t)P_3A3a_(t)(1/100α^2^+1/300α)+2P_4A2a_(t)P_2A4a_(t)(1/75α^2^+1/75α)

+2P_4A2a_(t)P_1A5a_(t)(1/90α^2^+1/30α)+2P_4A2a_(t)P_6a_(t)(1/15α)+P_3A3a_^2^(t)(1/400+9/400α^2^+3/200α)

+2P_3A3a_(t)P_2A4a_(t)(1/100+3/100α^2^+1/25α)+2P_3A3a_(t)P_1A5a_(t)(1/40+1/40α^2^+1/12α)

+2P_3A3a_(t)P_6a_(t)(1/20+3/20α)+P_2A4a_^2^(t)(1/25+1/25α^2^+2/25α)+2P_2A4a_(t)P_1A5a_(t)(1/10+1/30α^2^+2/15α)

+2P_2A4a_(t)P_6a_(t)(1/5+1/5α)+P_1A5a_^2^(t)(1/4+1/36α^2^+1/6α)+2P_1A5a_(t)P_6a_(t)(1/2+1/6α)+P_6a_^2^ (t)

**Supplemental Text 2: EM algorithm**

In what follows, we give a detailed procedure of implementing the EM algorithm to estimate parental-zygote frequencies without double reduction. Based on equation (1), the offspring-zygote frequencies without double reduction are derived and shown in equation (5). In the E step, the expected proportions of parental genotypes 6*A*, 5A1a, 4A2a, and 3A3a within an offspring genotype 6*A* are calculated as

$$\phi_{6A|6A}=P_{6A}(2P_{6A}+P_{5A1a}+\frac{2}{5}P_{4A2a}+\frac{1}{10}P_{3A3a})/R_{6A}$$

$$\phi_{5A1a|6A}=P_{5A1a}(P_{6A}+{\frac{1}{4}P}_{5A1a}+\frac{1}{5}P_{4A2a}+\frac{1}{20}P_{3A3a})/R_{6A}$$

$$\phi_{4A2a|6A}=P_{4A2a}(\frac{2}{5}P_{6A}+\frac{1}{5}P_{5A1a}+\frac{1}{25}P_{4A2a}+\frac{1}{50}P_{3A3a})/R_{6A}$$

$$\phi_{3A3a|6A}={2P}_{3A3a}(\frac{1}{10}P_{6A}+{\frac{1}{20}P}_{5A1a}+\frac{1}{100}P_{4A2a}+\frac{1}{400}P_{3A3a})/R_{6A}$$

The expected proportions of parental genotypes 6*A*, 5A1a, 4A2a, 3A3a, and 2A4a within an offspring genotype 5A1a are calculated as

$$\phi_{6A|5A1a}=(P_{5A1a}+\frac{6}{5}P_{4A2a}+\frac{9}{10}P_{3A3a}+\frac{2}{5}P_{2A4a})/R_{5A1a}$$

$$\phi_{5A1a|5A1a}=(P_{6A}+\frac{4}{5}P_{4A2a}+\frac{1}{2}P_{3A3a}+\frac{1}{5}P_{2A4a})/R_{5A1a}$$

$$\phi_{4A2a|5A1a}=(\frac{6}{5}P_{6A}+\frac{4}{5}P_{5A1a}+\frac{12}{25}P_{4A2a}^{2}+\frac{6}{25}P_{3A3a}+\frac{2}{25}P_{2A4a})/R_{5A1a}$$

$$\phi_{3A3a|5A1a}=(\frac{9}{10}P_{6A}+\frac{1}{2}P_{5A1a}+\frac{6}{25}P_{4A2a}+\frac{9}{100}P_{3A3a}^{2}+\frac{1}{50}P_{2A4a})/R_{5A1a}$$

$$\phi_{2A4a|5A1a}=(\frac{2}{5}P_{6A}+\frac{1}{5}P_{5A1a}+\frac{6}{25}P_{4A2a}+\frac{1}{50}P_{3A3a}+P_{2A4a}^{2})/R_{5A1a}$$

The expected proportions of parental genotypes 6*A*, 5A1a, 4A2a, 3A3a, 2A4a, and 1A5a within an offspring genotype 4A2a are calculated as

$$\phi_{6A|4A2a}=(\frac{2}{5}P_{6A}+\frac{9}{10}P_{3A3a}+\frac{6}{5}P_{2A4a}+P_{1A5a})/R_{4A2a}$$

$$\phi_{5A1a|4A2a}=(\frac{1}{2}P_{5A1a}^{2}+\frac{4}{5}P_{4A2a}+\frac{9}{10}P_{3A3a}+\frac{4}{5}P_{2A4a}+\frac{1}{5}P_{1A5a})/R_{4A2a}$$

$$\phi_{4A2a|4A2a}=(\frac{2}{5}P_{6A}+\frac{4}{5}P_{5A1a}+\frac{22}{25}P_{4A2a}^{2}+\frac{37}{50}P_{3A3a}+\frac{12}{25}P_{2A4a}+\frac{1}{5}P_{1A5a})/R_{4A2a}$$

$$\phi_{3A3a|4A2a}=(\frac{9}{10}P_{6A}+\frac{9}{10}P_{5A1a}+\frac{37}{50}P_{4A2a}+\frac{99}{200} P_{3A3a}^{2}+\frac{6}{25}P_{2A4a}+\frac{1}{20}P_{1A5a})/R_{4A2a}$$

$$\phi_{2A4a|4A2a}=(\frac{6}{5}P_{6A}+\frac{4}{5}P_{5A1a}+\frac{12}{25}P_{4A2a}+\frac{6}{25}P_{3A3a}+\frac{2}{25}P_{2A4a}^{2})/R_{4A2a}$$

$$\phi_{1A5a|4A2a}=(\frac{1}{2}P_{5A1a}+\frac{1}{5}P_{4A2a}+\frac{1}{20}P_{3A3a})/R_{4A2a}$$

The expected proportions of parental genotypes 6*A*, 5A1a, 4A2a, 3A3a, 2A4a, 1A5a, and 6a within an offspring genotype 3A3a are calculated as

$$\phi_{6A|3A3a}=(\frac{1}{10}P_{3A3a}+\frac{2}{5}P_{2A4a}+P_{1A5a}+2P_{6a})/R_{3A3a}$$

$$\phi_{5A1a|3A3a}=(\frac{1}{5}P_{4A2a}+\frac{1}{2}P_{3A3a}+\frac{4}{5}P_{2A4a}+P_{1A5a}+P_{6a})/R_{3A3a}$$

$$\phi_{4A2a|3A3a}=(\frac{1}{5}P_{5A1a}+\frac{12}{25}P_{4A2a}^{2}+\frac{37}{50}P_{3A3a}+\frac{22}{25}P_{2A4a}+\frac{1}{5}P_{1A5a}+\frac{2}{5}P_{6a})/R_{3A3a}$$

$$\phi_{3A3a|3A3a}=(\frac{1}{10}P_{6A}+\frac{1}{2}P_{5A1a}+\frac{37}{50}P_{4A2a}+\frac{41}{50}P_{3A3a}^{2}+\frac{37}{50}P_{2A4a}+\frac{1}{2}P_{1A5a}+\frac{1}{10}P_{6a})/R_{3A3a}$$

$$\phi_{2A4a|3A3a}=(\frac{2}{5}P_{6A}+\frac{4}{5}P_{5A1a}+\frac{22}{25}P_{4A2a}+\frac{37}{50}P_{3A3a}+\frac{12}{25}P_{2A4a}^{2}+\frac{1}{5}P_{1A5a})/R_{3A3a}$$

$$\phi_{1A5a|3A3a}=(P_{6A}+P_{5A1a}+\frac{4}{5}P_{4A2a}+\frac{1}{2}P_{3A3a}+\frac{1}{5}P_{2A4a})/R_{3A3a}$$

$$\phi_{6a|3A3a}=(2P_{6A}+P_{5A1a}+\frac{2}{5}P_{4A2a}+\frac{1}{10}P_{3A3a})/R_{3A3a}$$

The expected proportions of parental genotypes 5A1a, 4A2a, 3A3a, 2A4a, 1A5a, and 6a within an offspring genotype 2A4a are calculated as

$$\phi_{5A1a|2A4a}=(\frac{1}{20}P_{3A3a}+\frac{1}{5}P_{2A4a}+\frac{1}{2}P_{1A5a}+P_{6a})/R_{2A4a}$$

$$\phi_{4A2a|2A4a}=(\frac{2}{25}P_{4A2a}^{2}+\frac{6}{25}P_{3A3a}+\frac{12}{25}P_{2A4a}+\frac{4}{5}P_{1A5a}+\frac{6}{5}P_{6a})/R_{2A4a}$$

$$\phi_{3A3a|2A4a}=(\frac{1}{20}P_{5A1a}+\frac{6}{25}P_{4A2a}+\frac{99}{200}P_{3A3a}^{2}+\frac{37}{50}P_{2A4a}+\frac{9}{10}P_{1A5a}+\frac{9}{10}P_{6a})/R_{2A4a}$$

$$\phi_{2A4a|2A4a}=(\frac{1}{5}P_{5A1a}+\frac{12}{25}P_{4A2a}+\frac{37}{50}P_{3A3a}+\frac{22}{25}P_{2A4a}^{2}+\frac{4}{5}P_{1A5a}+\frac{2}{5}P_{6a})/R_{2A4a}$$

$$\phi_{1A5a|2A4a}=(\frac{1}{2}P_{5A1a}+\frac{4}{5}P_{4A2a}+\frac{9}{10}P_{3A3a}+\frac{4}{5}P_{2A4a}+\frac{1}{2}P_{1A5a}^{2})/R_{2A4a}$$

$$\phi_{6a|2A4a}=(P_{5A1a}+\frac{6}{5}P_{4A2a}+\frac{9}{10}P_{3A3a}+\frac{2}{5}P_{2A4a})/R_{2A4a}$$

The expected proportions of parental genotypes 4A2a, 3A3a, 2A4a, 1A5a, and 6a within an offspring genotype 1A5a are calculated as

$$\phi_{4A2a|1A5a}=(\frac{1}{50}P_{3A3a}+\frac{2}{25}P_{2A4a}+\frac{1}{5}P_{1A4a}+\frac{2}{5}P_{6a})/R_{1A5a}$$

$$\phi_{3A3a|1A5a}=(\frac{1}{50}P_{4A2a}+\frac{9}{100}P_{3A3a}^{2}+\frac{6}{25}P_{2A4a}+\frac{1}{2}P_{1A5a}+\frac{6}{10}P_{6a})/R_{1A5a}$$

$$\phi_{2A4a|1A5a}=(\frac{2}{25}P_{4A2a}+\frac{6}{25}P_{3A3a}+\frac{12}{25}P_{2A4a}^{2}+\frac{4}{5}P_{1A5a}+\frac{6}{5}P_{6a})/R_{1A5a}$$

$$\phi_{1A5a|1A5a}=(\frac{1}{5}P_{4A2a}+\frac{1}{2}P_{3A3a}+\frac{4}{5}P_{2A4a}+P_{1A5a}^{2}+P_{6a})/R_{1A5a}$$

$$\phi_{6a|1A5a}=(\frac{2}{5}P_{4A2a}+\frac{9}{10}P_{3A3a}+\frac{2}{5}P_{2A4a}+P_{1A5a})/R_{1A5a}$$

The expected proportions of parental genotypes 3*A*3*a*, 2*A*4*a*, 1*A*5*a*, and 6*a* within an offspring genotype 6*a* are calculated as

$\phi_{3A3a|6a}=P_{3A3a}$($\frac{1}{200}P_{3A3a}$+$\frac{1}{50}P_{2A4a}+\frac{1}{20}P_{1A5a}+\frac{1}{10}P_{6a}$)$/R_{6a}$

$$\phi_{2A4a|6a}={2P}_{2A4a}({\frac{1}{100}P}_{3A3a}+\frac{1}{25}P_{2A4a}+\frac{1}{10}P_{1A5a}+\frac{1}{5}P_{6a})/R_{6a}$$

$$\phi_{1A5a|6a}={2P}_{1A5a}({\frac{1}{40}P}_{3A3a}+\frac{1}{10}P_{2A4a}+\frac{1}{4}P_{1A5a}+\frac{1}{2}P_{6a})/R_{6a}$$

$$\phi_{6a|6a}={2P}_{6a}({\frac{1}{20}P}_{3A3a}+\frac{1}{5}P_{2A4a}+\frac{1}{2}P_{1A5a}+P_{6a})/R_{6a}$$

In the M step, we calculate parental-zygote frequencies based on the results from the M step by

$$R_{6A}=\frac{1}{2N}(N_{6A}\phi_{6A|6A}+N_{5A1a}\phi_{6A|5A1a}+N_{4A2a}\phi_{6A|4A2a}+N_{3A3a}\phi_{6A|3A3a})$$

$$R_{5A1a}=\frac{1}{2N}(N_{6A}\phi_{5A1a|6A}+N_{5A1a}\phi_{5A1a|5A1a}+N_{4A2a}\phi_{5A1a|4A2a}+N_{3A3a}\phi_{5A1a|3A3a}+N_{2A4a}\phi_{5A1a|2A4a})$$

$$R_{4A2a}=\frac{1}{2N}(N_{6A}\phi_{4A2a|6A}+N_{5A1a}\phi_{4A2a|5A1a}+N_{4A2a}\phi_{4A2a|4A2a}+N_{3A3a}\phi_{4A2a|3A3a}+N_{2A4a}\phi_{4A2a|2A4a}+N_{1A5a}\phi_{4A2a|1A5a})$$

$$R_{3A3a}=\frac{1}{2N}(N_{6A}\phi_{3A3a|6A}+N_{5A1a}\phi_{3A3a|5A1a}+N_{4A2a}\phi_{3A3a|4A2a}+N_{3A3a}\phi_{3A3a|3A3a}+N_{2A4a}\phi_{3A3a|2A4a}+N_{1A5a}\phi_{3A3a|1A5a}+N_{6a}\phi_{1A5a|6a})$$

$$R_{2A4a}=\frac{1}{2N}(N_{5A1a}\phi_{2A4a|5A1a}+N_{4A2a}\phi_{2A4a|4A2a}+N_{3A3a}\phi_{2A4a|3A3a}+N_{2A4a}\phi_{2A4a|2A4a}+N_{1A5a}\phi_{2A4a|1A5a}+N_{6a}\phi_{2A4a|6a})$$

$$R_{1A5a}=\frac{1}{2N}(N_{4A2a}\phi_{1A5a|4A2a}+N_{3A3a}\phi_{1A5a|3A3a}+N_{2A4a}\phi_{1A5a|2A4a}+N_{1A5a}\phi_{1A5a|1A5a}+N_{6a}\phi_{1A5a|6a})$$

$$R_{6a}=\frac{1}{2N}(N_{3A3a}\phi_{6a|3A3a}+N_{2A4a}\phi_{6a|2A4a}+N_{1A5a}\phi_{6a|1A5a}+N_{6a}\phi_{6a|6a})$$

The E- and M-steps are iterated until the estimates converge to stable values. These stable values are the MLEs of genotype frequencies in the parent population under the assumption of no double reduction.
